# A carbon nanotubes based in situ multifunctional power assist system for restoring failed heart function

**DOI:** 10.1186/s42490-021-00051-x

**Published:** 2021-03-26

**Authors:** Quanfu Xu, Yuli Yang, Jianwen Hou, Taizhong Chen, Yudong Fei, Qian Wang, Qing Zhou, Wei Li, Jing Ren, Yi-Gang Li

**Affiliations:** 1 Department of Cardiology, Xinhua Hospital, School of Medicine, Shanghai Jiao Tong University, Shanghai, 200092 China; 2 School of Physical Science and Technology, ShanghaiTech University, 393 Middle Huaxia Road, Shanghai, 201210 China

**Keywords:** Aligned materials, Carbon nanotubes, Heart failure, Cardiac pacing, Power assist device

## Abstract

**Background:**

End-stage heart failure is a major risk of mortality. The conductive super-aligned carbon nanotubes sheets (SA-CNTs) has been applied to restore the structure and function of injured myocardium through tissue engineering, and developed as efficient cardiac pacing electrodes. However, the interfacial interaction between SA-CNTs and the surface cells is unclear, and it remains challenge to restore the diminished contraction for a seriously damaged heart.

**Results:**

A concept of a multifunctional power assist system (MPS) capable of multipoint pacing and contraction assisting is proposed. This device is designed to work with the host heart and does not contact blood, thus avoiding long-term anticoagulation required in current therapies. Pacing electrode constructed by SA-­CNTs promotes the epithelial-mesenchymal transition and directs the migration of pro-regenerative epicardial cells. Meanwhile, the power assist unit reveals an excellent frequency response to alternating voltage, with natural heart mimicked systolic/diastolic amplitudes. Moreover, this system exhibits an excellent pacing when attached to the surface of a rabbit heart, and presents nice biocompatibility in both in vitro and in vivo evaluation.

**Conclusions:**

This MPS provides a promising non-blood contact strategy to restore in situ the normal blood-pumping function of a failed heart.

**Supplementary Information:**

The online version contains supplementary material available at 10.1186/s42490-021-00051-x.

## Background

End-stage congestive heart failure, characterized by ventricular dilation, diffusely reduced myocardial contractility and frequently ventricular dyssynchrony, is the last outcome of various heart diseases [1, 2]. Among them, heart failure developed from myocardial infarction are soaring and has become a major source of mortality [3, 4]. Cardiac transplantation, as the only definitive solution, is yet limited by organ shortage and adverse effects of lifelong immunosuppressive therapies [5, 6]. The currently available mechanical circulatory support devices are mainly used as a bridge to heart transplantation. Because of their contact with blood and requirement of long-term anticoagulant, these devices can lead to various serious complications such as bleeding and thrombosis etc. [7–10]. Cardiac tissue engineering is a promising option to in situ restore the infarcted heart and its deteriorating function while preserving the host’s heart [11–14]. In our previous study, conductive super-aligned carbon nanotubes sheets (SA-CNTs) induced a natural myocardium mimicked morphology of cultured cardiomyocytes and provided efficient extracellular signal transmission pathways [15]. SA-CNTs based one-piece electrodes performed an excellent multipoint pacing function through direct attachment on heart surface, which provides potential applications in cardiac resynchronization therapy [15].

However, the primary study and many other cardiac engineering related researches mostly focused on cardiomyocytes, whose morphology and electrophysiology can be significantly influenced by the material/cells interfaces [16–18]. In fact, the outermost layer of natural heart is the residence of epicardial cells, which differ from cardiomyocytes in morphology and physiological function [19]. Previous reports including our own data have shown that epicardial cells play a crucial role in heart development and cardiac repair [19–21]. Through epithelial-mesenchymal transition (EMT), they provide the progenitors (epicardial-derived progenitor cells, EDPCs) for coronary vascular mural cells and cardiac fibroblasts, and to a lesser extent, endothelial cells and cardiomyocytes [19, 22]. It remains unclear how the as developed SA-CNTs-based pacing system and electrical impulse would affect the epicardial cells.

On the other hand, as a dynamic muscular organ, heart is responsible for pumping blood throughout the body by powerful bi-ventricular synchro contraction. The occurrence and progression of heart failure result in diminished myocardial contractility and asynchronous contractions of two ventricles, which seriously affect the pumping efficiency of heart [2, 23]. Heart failure, especially end-stage heart failure is currently unable to be fully recovered by pharmacological and device therapy including cardiac resynchronization therapy [24, 25]. Therefore, by endowing the cardiac resynchronization pacing electrode with electric-driven power assist function, it could provide great potential to restore the disordered biventricular synchrony as well as impaired myocardial contractility in a failed heart.

**Fig. 1:**
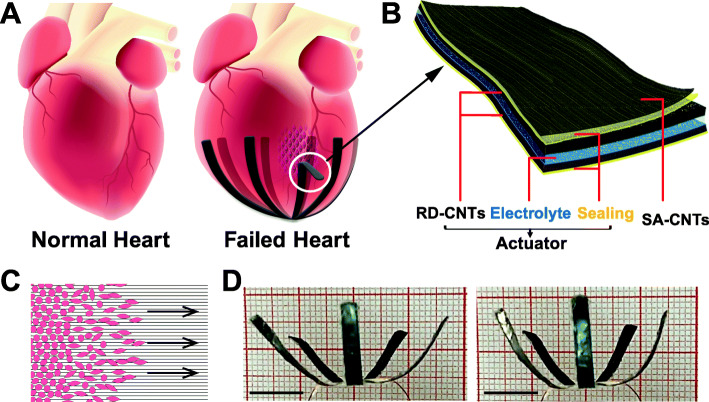
Schematic illustration demonstrating the CNTs based multifunctional power assist system (MPS). **a** Schematic illustration of a normal heart and application of the MPS for a dilated and failed heart. **b** Microstructure of electric driven multifunctional power assist device. **c** Schematic indicating SA-CNTs guide the migration of epicardial cells. **d** Representative images showing MPS in diastolic (left) and systolic state (right). Scale bars, 1 cm. RD-CNT, randomly dispersed carbon-nanotube film; SA-CNTs, superaligned carbon-nanotube sheets. Electrolyte, polyvinyl alcohol (PVA) gel filled with sulfuric acid; Sealing, poly (dimethylsiloxane) (PDMS).

Herein, we present a carbon nanotubes (CNTs) based and heart beating mimicked multifunctional power assist system (MPS). By enveloping heart in situ, the MPS provides a multipoint pacing function as well as an electric-driven power-assist function for a failed heart in a non-blood contact manner (Fig. 1a and b). Meanwhile, as inner layer of MPS contacting the heart surface, the SA-CNTs provide a unique interface to induce directed migration and enhanced EMT of epicardial cells (Fig. 1c), which are routinely observed in cardiac repair and regeneration after injure. By enveloping a heart, the MPS 1) offers the potential to repair the injured myocardium through activating and enhancing the regeneration activities of epicardial cells, 2) provides synchronous electrical pacing signals to two ventricles with disordered synchronization, and 3) provides addition systolic-diastolic power assist to myocardium with impaired contractility. Thus, the MPS shows triple efforts to help a failed heart to restore normal blood-pumping function and would be promising in the treatment of patients with heart failure, especially in heart failure patients after myocardial infarction.

## Methods

### Preparation of the SA-CNTs-based flexible pacing electrode

Same as our previous work, SA-CNTs were obtained by dry spinning process. SA-CNTs were directly pulled from spinnable CNT forests and laid on a Coverglass or a thin poly (dimethylsiloxane) (PDMS) substrate [15]. The spinnable CNT forest was synthesized by a chemical vapor deposition method. SA-CNTs laid on a Coverglass was used for in vitro cell culture, whereas SA-CNTs laid on a thin PDMS substrate was applied as a flexible electrode for pace-making. The samples were placed in 35-mm diameter plastic culture dishes or 6-well plates and sterilized by 60-min exposures to UV radiation in a biosafety cabinet.

### Preparation of the power assist unit

Electrochemical actuator petals were firstly assembled. Ions filled gel electrolyte was prepared by dissolving 1.5 g polyvinyl alcohol (PVA, molecular weight: 2000–4000) in 8.5 g deionized water at 90 °C (swelling at 40 °C for 3 h before dissolving). After cooling down by ice bath, 1 g sulfuric acid (98%) was added into the PVA gel under stirring. Commercially purchased randomly dispersed CNTs-film (RD-CNT, JERNANO, Suzhou, China) was cut into small strips (e.g., 3 × 20 mm^2^). Gel electrolyte was sandwiched between two CNTs-strips and dried in the air (room temperature of 20 °C, relative humidity of 45%). After 2 days, the water content of the gel was below 1% and remained unchanged at this condition. Five or more as prepared actuator petals were then assembled into a flower-shape, connected to external power source by copper wire and silver paint, and packed with PDMS for sealing.

### Characterizations of the electrode materials and device structures

The morphology of the CNTs materials and structure or the device were characterized by scanning electron microscopes (SEM, JSM-7800MF). The resistance of the CNTs film was measured using a Keithley Model 2400 Source Meter. The mechanical property of the electrochemical actuator petal was measured by Instron 5966 machine (Norwood, USA).

### Embryonic epicardial ex vivo culture and characterizations

Primary epicardial cells were obtained by outgrowth culture assay from embryonic mouse hearts and embryonic cell was cultured as previously reported [26]. Briefly, embryos were taken from pregnant wild type C57Bl/6 mice at E12.5. E12.5 hearts were dissected and the ventricles were plated with the epicardial side onto SA-CNTs or Coverglass (WHB Biotechnology Co., Ltd., CHN) in Dulbecco’s modified eagle medium (DMEM), supplemented with 100 units/ml Penicillin, 100 μg/ml Streptomycin (Gibco) and 10% fetal bovine serum (FBS,10091–148, Gibco, Grand Island, NY, USA), and placed in a 37 °C, 5% CO_2_ humidified incubator. A blank Coverglass was used as a control. Epicardial cells live in the outermost layer of heart. When embryonic mouse hearts were plated with the epicardial side onto substrates, epicardial cells at the heart surface/substrate interface migrated and expanded spontaneously from the heart to the surface of the substrate as a result of epithelial spreading.

Electrical stimulation was applied from 48 h after seeding by directly connecting the conductive SA-CNTs with clinical pace-makers (rectangular, 2 ms, 2 V cm^− 1^, 1 Hz) (Model 5318, Medtronic, Inc., USA). The connecting parts were protected by insulated PDMS to avoid electric leakage, thus the pacing pulse would reach cells through the conductive substrate.

Epicardial cells are identified with characteristic mesothelial marker (nuclear Wilms-tumor1, WT1) and organized epithelial tight junctions (Zonula occludens-1, ZO1). Mesenchymal cells were defined by loss of the epithelial type of ZO1 expression and gain of representative mesenchymal marker α-smooth muscle actin (α-SMA) and cellular morphology. All animal experiments were approved by the Animal Care and Use Committee of Shanghai Xinhua Hospital affiliated to Shanghai Jiao Tong University School of Medicine.

### Power assist unit assessment

Electrochemical performance of the as assembled electrochemical actuator petal was tested by Cyclic Voltammetry (CV) method through a CHI 660C electrochemical workstation. Meanwhile, deformation of the electrochemical actuator was driven by the cyclic alternating voltage provided by the CV test with controlled scan rates (1 to 25 V s^− 1^) and voltage window (e.g., + 2.0 to − 2.0 V, + 2.5 to − 2.5 V). Electrochemical performance of CNTs films was also tested by the three-electrode method, with Ag/AgCl as reference electrode and Pt as counter electrode in an 10% H_2_SO_4_ aqueous electrolyte. Deformation frequency and magnitude of the flower-shaped power assist unit was evaluated by recording the process in situ (iPhone 6, 60 frame per second), and then analyzed and diagrammed frame by frame using ImagePro Plus software.

### Simulation method

The finite element method simulation for flower-shaped power assist unit shown in Fig. S16 (1 V S^− 1^, Supporting Information) were performed to evaluate the stress distribution along the device. The model was built in Hypermesh and the strips was simulated using the S4R shell element. Material and unit definitions were done in ABAQUS. The actuator petal was considered as an isotropic material with a density of 1 × 10^− 8^ kg/mm^3^, Young’s modulus of 334 MPa, Poisson’s ratio of 0.26 and a thickness of 50 μm. The node displacement function before and after the contraction was calculated by the ellipse formula, and the local coordinate system of each strip was defined accordingly. The space field function was defined under the same conditions. The field function was introduced to the node displacement load. The movement of each petal at the long axis direction was constrained. According to the movement behavior of the flower-shaped power assist unit recorded in Video S1 (Supporting Information), the displacement field at the nodes was defined: the maximum displacement was 3 mm. Before contraction, the long axis was 36 mm and the short axis was 33 mm. After contraction, the long axis was 36 mm and the short axis was 27 mm. Boundary condition: the vertical degree of freedom of the joint nodes of each strip was constrained. Implicit time method for history analysis was used with a calculation time of 1 s.


**Video S1.** Video of a flower-shaped power assist unit performing heart mimicked systolic-diastolic behavior with increased scan rates at voltage window of ± 2.5 V.

### Cardiac pacing assessment

Cardac pacing was evaluated using Langendorff perfusion system. After intravenous anesthetization with sodium pentobarbital (30 mg/kg), rabbit hearts were immediately removed and mounted on a Langendorff apparatus and perfused through the aorta (30 mL/min) with Tyrode’s solution (in mM: 135 NaCl, 5 KCl, 1 MgCl_2_, 1.8 CaCl_2_, 10 HEPES, and 10 glucose, adjusted to pH 7.4 with NaOH). The electric drive power assist device was placed directly onto the surface of rabbit heart and connected to a pacemaker (Medtronic, Inc., USA) and paced at rectangular pulses, 2 ms, 5 V, 100 bpm (beat per minute). During long-term testing, the distal end of the flower-shaped actuator needs to be adhered to the surface of the heart with bio-glue. Continuous ECG monitoring was recorded and analyzed using Spike 2 (Cambridge Electronic Design, Ltd., Cambridge, UK) and Origin Pro 8.6 software (OriginLab Corporation, Northampton, MA, USA).

### In vivo degradation and biocompatibility of MPS

C57bl/6 male mice were used in in vivo studies. All animal experiments were approved by the Institutional Animal Care and Use Committee of Xinhua Hospital in accordance with the Guide for the Care and Use of Laboratory Animal published by the National Institutes of Health (Approval No, XHEC-F-2018-054). A 1 cm incision in the mediodorsal skin of was made and a lateral subcutaneous pocket prepared. MPS samples (0.5 × 1 cm) were implanted under sterile conditions. The mice were sacrificed and the tissue samples were processed for histological staining at 2 weeks and 4 weeks after implantation to evaluate biodegradation and biocompatibility.

### Statistical analysis

Data are presented as the means ± standard error of means unless otherwise indicated. Differences between two groups were evaluated using Student’s t-test or chi-squared tests as appropriate. Multiple comparisons were measured by one-way ANOVA followed by a Bonferroni post hoc test. Tests for trends (*p* for trend) was calculated with Logistic regression. Probability values < 0.05 were considered significant, and 2-sided tests were performed.

## Results and discussion

### Design and fabrication of MPS

The MPS consists of two main parts: a SA-CNTs-based flexible pacing electrode in direct contact with the surface of the heart, and a CNTs-film-based power assist unit packed in nontoxic PDMS (Fig. 1b and d). The power assist unit is an electrical driven electrochemical actuator consisting of two CNTs-film electrodes and an ions-filled polymer gel electrolyte. CNTs are widely explored for the wearable and implantable devices due to their unique structure, excellent mechanical property, large surface area, excellent charge transport capability and tunable morphology [27–30]. The SA­CNTs in the pacing electrode part were prepared by dry­spinning process from a spinnable CNT forest, which was synthesized via chemical vapor deposition (CVD) method [31]. SA­CNTs show a highly aligned morphology, where CNTs tend to form CNT bundles due to the Van der Waals’ force, thus leaving nano-scaled free space among them (Fig. S1, Supporting Information) and can offer an anisotropic interface between SA­CNTs and contacted cells.

### Biocompatibility and interaction between SA-CNTs and Epicardial cells

Biocompatibility is essential to developing implantable device. The MPS was mostly packaged in biocompatible PDMS [32, 33], except for the SA-CNTs based electrode, which act as the interface between MPS and heart and is directly contacting the epicardium of a heart. Therefore, interfacial interactions between SA-CNTs and cardiac epicardial cells were firstly investigated. In our previous study, besides an excellent biocompatibility, SA­CNTs demonstrated a function of guiding the orientation and extension of cardiomyocytes, and showed an efficient transmitting of electrical signal for the cultured cardiac tissue in vitro [15]. In this work, the effect of SA-CNTs on epicardial cells was investigated by seeding primary epicardial cells onto the SA-CNTs and compared with those cells seeding on the Coverglass under the same condition. Primary epicardial cells were obtained by outgrowth culture from embryonic mouse hearts [26]. By planting an embryonic mouse heart (E 12.5) on the substrate and culturing in vitro, epicardial cells at the heart surface/substrate interface migrated and expanded spontaneously from the heart to the surface of the substrate as a result of epithelial spreading (Fig. S2, Supporting Information) [26]. Epicardial cells were identified with characteristic mesothelial marker (WT1) and organized epithelial tight junctions (ZO1) (Fig. S3, Supporting Information). After a certain amount of epicardial cells had reached to the substrate, the original embryonic mouse heart can be removed, and the morphology and physiologic properties of these epicardial cells on the substrates were further evaluated.

**Fig. 2:**
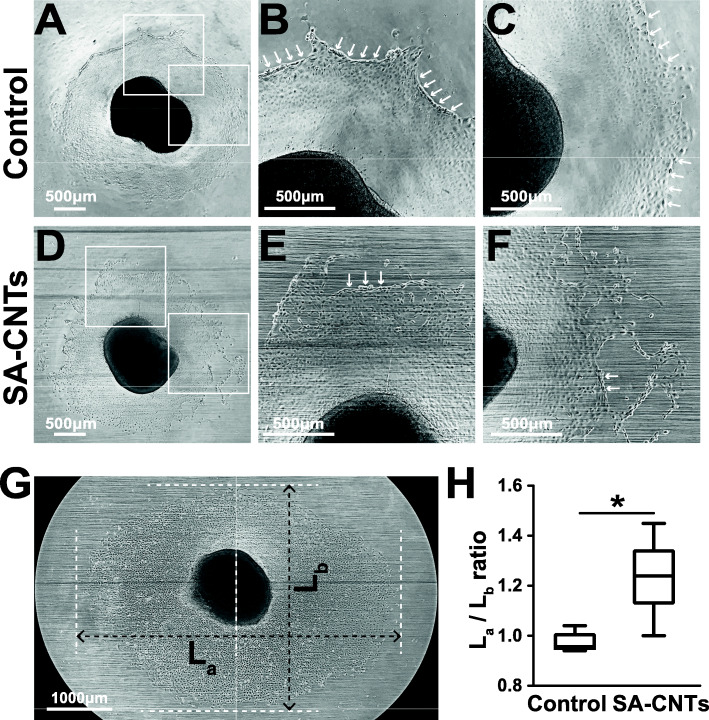
SA-CNTs facilitate epicardial cells outgrowth from embryonic mouse hearts and guide the migration direction of outgrowing epicardial cells. **a**-**f** Representative phase contrast light microscopy images of epicardial outgrowth at Day3 indicating peripheral cells on Coverglass resulted in a formation of “Dam” (indicated with white arrow) which hinders the central cells from moving forward until the cells break through the dam (**a**-**c**), whereas this phenomenon rarely observed in outgrowing cells on SA-CNTs (**d**-**f**). **b**, **c** The enlarged image of a selected zone in (**a**); (**e**, **f**) The enlarged image of a selected zone in (**d**). **g** Typical image (stitched from two fields of one heart explant) after 4 day of culture showing that epicardial cells on SA-CNTs tend to migrate along the CNT-aligned direction. The maximum transverse and longitudinal migration distance of cells were measured as L_a_ and L_b_ using ImageJ and shown as L_a_/L_b_ ratio in (**h**). **h** Migration pattern indicated using L_a_/L_b_ ratio (divide L_a_ by L_b_) showing that epicardial cells on SA-CNTs have its own unique migration manner due to the superaligned structure of SA-CNTs. *n* = 7 for control sample and *n* = 13 for SA-CNTs from 5 independent experiments. *p < 0.05. Scale bars, 500 μm

Epicardial outgrowth from the original embryonic mouse heart to both Coverglass and SA-CNTs were observed within 24 h after explant planting (Fig. S4, Supporting Information). The epicardial outgrowth primarily consisted of mesothelial cells with cobblestoned morphology, which is consistent with previous reports [21, 26]. The cell outgrowth was then traced and recorded during 4 days of incubation. At Day2, epicardial outgrowth on Coverglass (control) formed a tightly packed sheet of epithelial cells, while cells on SA-CNTs begun to exhibit a lamellipodia and migratory behavior, especially for those peripheral cells in the epithelial sheets (Fig. S5, Supporting Information). At day3, the outermost cells from the center heart on Coverglass seemed stuck to the substrate and stopped moving forward, resulting in a newly formed “Dam”. The latter outgrowing cells would be trapped in this “Dammed lake”, until the trapped cells accumulated to an extent that exceed the capacity of the “Dam”, they broke through the embankment and pour out (Fig. 2a-c and S6, Supporting Information).

In comparison, the migration behavior of peripheral cells on SA-CNTs was quite different, because the surface provided by SA-CNTs is anisotropic. Firstly, the formation of cellular “Dam” was rarely observed on SA-CNTs (Fig. 2d-f). Secondly, when the epicardial cells on Coverglass migrated around in a non-directional manner (L_a_ = L_b_, Fig. S7, Supporting Information), the cells on SA-CNTs tended to migrate more along the orientation of the SA-CNTs (L_a_ > L_b_, Fig. 2g). Therefore, the migration distance of epicardial cells in the SA-CNTs aligned direction is longer than that in the vertical direction (Fig. 2h). The SA-CNTs is ultra-thin (around 20 nm) compared to the micrometer-scaled cells, therefore the CNT bundles showed no obvious hindrance to cell migration at the vertical direction. However, the SA-CNTs promoted cell migration at the orientation direction, which was corresponding to their guiding function of elongated cell morphology for cultured cardiomyocytes in our pervious study [15]. This cell migration guidance phenomenon was confirmed by quantitative measurements (Fig. 2h and S8, Supporting Information). These results suggest that by controlling the physical interface interactions, which is the aligned morphology of CNTs in this work, more pro-regenerative epicardial cells can be directed to the infarcted area to participate in cardiac repair.

On the other hand, the SA-CNTs-based flexible pacing electrode is expected to provide electrical pacing signals to the contacted heart. So, continuous electrical impulse (rectangular, 2 ms, 2 V cm^− 1^, 1 Hz) was applied to the epicardial cells by connecting the conductive SA­CNTs to a pacemaker. The electrical stimulation started at 48 h after the heart planting onto the substrates, when the epicardial outgrowth had become relatively stable. Results showed that the cell migration behavior with electrical stimulation was similar to that observed on SA-CNTs without stimulation (Fig. S9, Supporting Information).

Moreover, the biocompatibility of SA-CNTs was also evaluated by detecting the cytotoxicity of SA-CNTs with/without electrical stimulation using TUNEL (TdT-mediated dUTP nick-end labeling) staining of apoptotic cells. Both the SA-CNTs with and without electrical stimulation showed no significant cytotoxicity to the epicardial cells compared to the Coverglass (Fig. S10, Supporting Information).

**Fig. 3:**
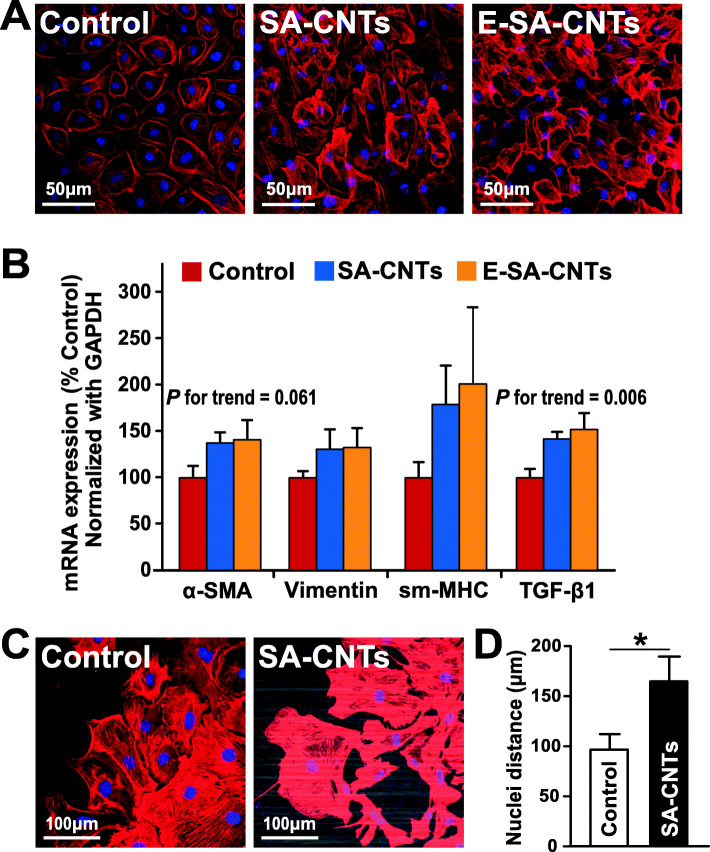
SA-CNTs promote EMT in outgrowing epicardial cells. **a** Representative confocal images indicating that SA-CNTs (mid) and SA-CNTs plus external electrical stimulation (E-SA-CNTs, right) increased the expression of EMT marker α-SMA (red) compared with control (left) after 6 day of culture by immunofluorescent staining of the central cells of the epicardial outgrowth. **b** mRNA expression of EMT marker α-SMA, Vimentin, sm-MHC and EMT inducer TGF-β1 after 6 day of culture in SA-CNTs and E-SA-CNTs groups. *n* = 6 for each group from 4 independent experiments. Tests for trends (p for trend) was calculated with Logistic regression. **c** Representative confocal images indicating that epicardial cells grown out from heart explant placed on SA-CNTs at 6 day of culture exhibited an enhanced migration indicated by greater separation of nuclei and increased presence of lamellipodia. **d** Quantitation of cell separation (indicated by nuclei distance) of epicardial cells grown out from heart explant on control and SA-CNTs after 6 day of culture. *n* = 5 for control sample and n = 5 for SA-CNTs from 5 independent experiments. *p < 0.05

The morphological and behavioral changes observed in epicardial cells on SA-CNTs suggest that the cells on SA-CNTs may undergo EMT. EMT is a biological process, in which epithelial cells lose cell polarity and epithelial phenotypes, transform into mesenchymal phenotype cells, and obtain higher interstitial phenotypes such as migration and invasion [34, 35]. To verify this assumption, expressions of EMT markers of outgrowing cells on SA-CNTs and Coverglass were assessed by immunocytochemistry and quantitative real-time polymerase chain reaction (qRT-PCR). Immunostaining of α-smooth muscle actin (α-SMA) indicated that its expression was increased in outgrowing epicardial cells on SA-CNTs, and further slightly enhanced after external electrical stimulation (Fig. 3a and b). qRT-PCR was also performed targeting on EMT markers α-SMA, Vimentin and Smooth muscle myosin heavy chain (SM-MHC), and EMT inducer Transforming growth factor (TGF)-β1. For these epicardial cells, SA-CNTs increased significantly the mRNA expression of EMT inducer TGF-β1 (Fig. 3b). The mRNA expression of α-SMA, SM-MHC and Vimentin were also increased in SA-CNTs and E-SA-CNTs group, but the difference was not statistically significant (Fig. 3b). Moreover, corresponding to the above microscopy observation, immunostaining results indicated that the SA-CNTs increase the cells in lamellipodia, and enhance the cell separation (a significant increase in distance between nuclei, Fig. 3c and d), which further confirmed the enhanced cell migration and the accelerated EMT.

### Performance and mechanism of power assist unit

The second part of the MPS is a power assist unit, which is directly attached onto the other side of the flexible pacing electrode. The power assist unit is an electrical driven actuator, providing assisted systolic-diastolic force along with the required synchronous electrical impulses for the seriously damaged heart. In this work, a flower-shaped MPS was designed to envelop the apical part of the heart (where frequently develop akinesis or even dyskinesis due to infarction expansion [36, 37]) and provide centripetal contractions-relaxation (Fig. 1a and d). The shape and size of the MPS can be tailored to suit the target heart and envelop the desired area (Fig. S11, Supporting Information). The electrical driven actuator was assembled by sandwiching an ions-filled polymer electrolyte between two flexible CNTs-film electrodes and packed in nontoxic PDMS (Fig. 1b). Each actuator petal was light-weight (0.3 × 2 cm^2^, 3.6 mg) with a thickness of around 50 μm (Fig. S12, Supporting Information). Different from the SA-CNTs in the pacing electrode, CNTs-film was commercially produced with a thickness of 5–10 μm, where the CNTs was randomly dispersed (Fig. S13, Supporting Information). This film showed excellent conductivity and flexibility (3.0 × 10^4^ S m^− 1^, 60–120 MPa), which can meet the demands of an actuator at low cost.

**Fig. 4:**
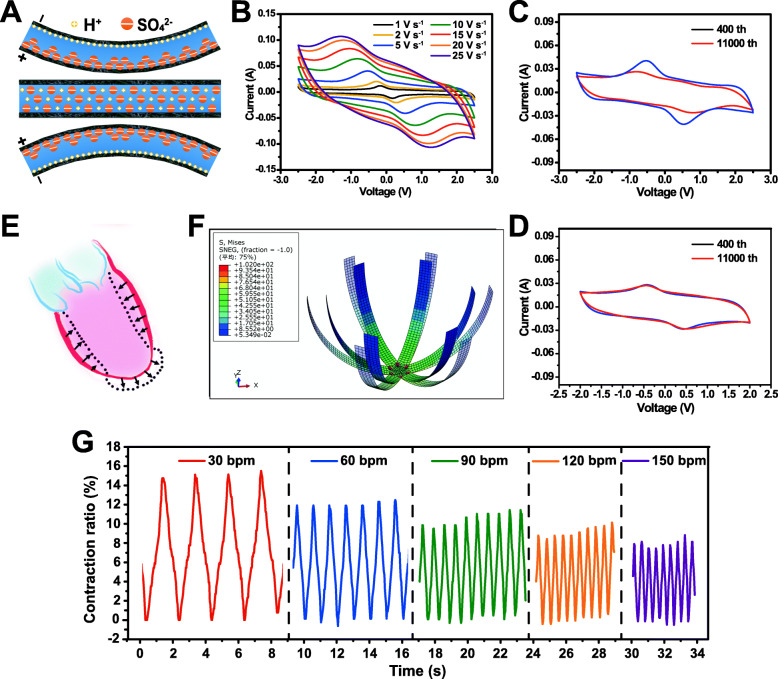
Mechanism and performance of the power assist unit. **a** Electrochemical actuation mechanism of the power assist unit. **b** Typical CV curves of one actuator petal at different scan rates. **c**, **d** Representative cyclic performance of an actuator petal with voltage window of (**c**) ± 2.5 V and (**d**) ± 2.0 V at the scan rate of 5 V s^− 1^, respectively. **e** Illustration of the systole and diastole of a failed heart post-infarction. Dotted line represents the systolic state and demonstrates the infarcted area with dyskinesis and bulging. The arrow indicates the direction of myocardial movement during contraction. **f** Stress contribution map of the power assist unit conducted by computer simulation. **g** Contraction performance of the power assist unit under increased voltage change frequency: 5, 10, 15, 20 and 25 V s^− 1^, corresponding to a heart systolic-diastolic behavior of 30, 60, 90, 120 and 150 bpm

The working principle of the electrochemical actuator petal is based on the electric double layer phenomenon [38–41]. When potential difference was applied at the two electrodes, ions (positive: H^+^, negative: SO_4_^2−^) in the gel electrolyte run towards their corresponding electrodes to balance the internal electric field. Due to the different volume of positive and negative ions, their directional movement and accumulation led to asymmetric volume changes near these two electrodes, consequently resulting in a deformation of the actuator petal (Fig. 4a). When the voltage of external circuit reversed, the actuator petal bent to the opposite direction. A petal in deformation can lift an object ≈ 4.7 times of its own weight and push an object ≈ 35.3 times of its own weight, which is similar to that of an adult human heart (250-350 g) outputting a force of ≈ 5.90 times of its weight (Fig. S14, Supporting Information) [42, 43]. Therefore, the heart beating mimicked systolic-diastolic behavior can be realized through cyclic alternating voltage.

Alternating voltage at different frequency was provided by CV method (Electrochemical workstation) with different scan rates. The as fabricated electrochemical actuator is also an electrochemical supercapacitor [40, 41, 44, 45], which was indicated by the CV curves (Fig. 4b). The device showed a typical supercapacitor behavior at increased scan rates from 1 to 25 V s^− 1^ with the potential range of − 2.5 V to + 2.5 V or − 2.0 V to + 2.0 V (Fig. 4b, and S15, Supporting Information). The specific capacitance (Csp) based on the CV curves was calculated with:

$$ {C}_{sp}=\frac{1}{2\bullet s\bullet m\bullet \Delta  V}{\int}_{V0}^{V0+\Delta  V} idV $$

Where *i* is current and *V* is potential, ∆*V* is potential range, *s* is scan rate, and *m* is mass of the whole device. *Csp* was 1.3 F g^− 1^ at low scan rate of 1 V s^− 1^, and was 0.7 F g^− 1^ at high scan rate of 25 V s^− 1^ (− 2.5 V to + 2.5 V). The shape of CV curves was mostly maintained at high scan rates, indicating a fast response capability.

The calculated Csp decreased to 0.6 g^− 1^ at low scan rate of 1 V s^− 1^, and 0.4 F g^− 1^ at high scan rate of 25 V s^− 1^ when the potential range narrowed to − 2.0 V to + 2.0 V, which was also corresponded to the actuator’s systole-diastole mimicked behavior. Here, contraction ratio (CR) was defined as (end diastolic dimension – end systolic dimension)/end diastolic dimension× 100%. The CR of a flower-shaped power assist unit was 19.4% when under the operation voltage range of − 2.5 V to + 2.5 V, and was 9.4% when under the operation voltage range of − 2.0 V to + 2.0 V (Fig. S16, Supporting Information). Although the CR decreased with the increased potential scan rate, the CR at 25 V s^− 1^ still remained 58% of that at 5 V s^− 1^ (Fig. S17, Supporting Information).

On the other hand, the electrolyte prepared here involved a small amount of water (lower than 10% weight percentage), while the industry produced CNTs films contained some metal catalyst (e. g., Fe, Ni, 5–15% weight percentage). Therefore, both the CV test of the assembled device or the three-electrode electrochemical testing system (CNTs film as working electrode v.s. Ag/AgCl electrode, Fig. 4c-d, S18, Supporting Information) revealed an irreversible electrode redox reaction. The irreversible reaction tent to be more noticeable at the wider operation voltage window, but was reduced after 10,000 cyclic scan process (Fig. 4c). Meanwhile, no significant change was observed during 10,000 cyclic operations at − 2.0 V to + 2.0 V (Fig. 4d).

Contraction behavior of the power assist unit was further evaluated by finite element method analysis. In fact, a normal heart contracts in a centripetal manner when bumping blood. However, for a failed heart post-infarction, the infarcted area is replaced by a thin fibrous scar and thus lost the ability to contract, or even exhibits dyskinesis and bulges out during systole; while the non-infarcted area undergoes remodeling, thinning and develops hypokinesis (Fig. 4e). The computer simulated stress distribution map and structure deformation map indicated that the bottom of the flower-shaped device has a larger stress with a smaller deformation, while the top part of each petal reveals a small stress with large position shifting (Fig. 4f and S19, Supporting Information). This result suggests that the flower-shape design may offer a structure restriction to the thin scar area while providing additional inward force to the myocardium with reduced contractility. On the other hand, the Young’s modulus of the actuator petal prepared in this work was 334 ± 11 MPa (Fig. S20, Supporting Information), which can be tuned and further influences the stress distribution of the device. Thus, the inward force for the failed myocardium can be enhanced by increasing the Young’s modulus of the actuator petals (Fig. S21, Supporting Information).

In general, the actuator’s systole-diastole mimicked behavior was controlled by the alternating voltage. 1) The power assist unit always showed a fast responsiveness when the voltage change frequency accelerated from 5, 10, 15, 20 to 25 V s^− 1^, corresponding to the heart beat of 30, 60, 90, 120, and 150 beat per minute (bpm) (Fig. 4g, Video S1, Supporting Information), which far covered the normal heart rate range (60–100 bpm); 2) The contraction amplitude (expansion dimension minus contraction dimension) decreased 10% when the voltage change frequency accelerated 25 times or the operation voltage window decreased from ±2.5 V to ±2.0 V. This phenomenon is similar to the case in human, when the heart rate goes up, the ejection fraction decreases. For patients with heart diseases, an increase in heart rate from 83 bpm to 154 bpm results in 31% reduction in ejection fraction [46]. It is worth noting that one aim of this work is to explore the feasibility of our design, such as the matchability between the stress and deformation distribution, and the response frequency of the flower-shaped electrochemical driven actuator compared with organs. By learning from the achievement of other works, optimized actuator with stronger contraction force and safer electrolyte can be further explored [38, 47, 48].

### Application and biostability of the MPS in vivo

**Fig. 5:**
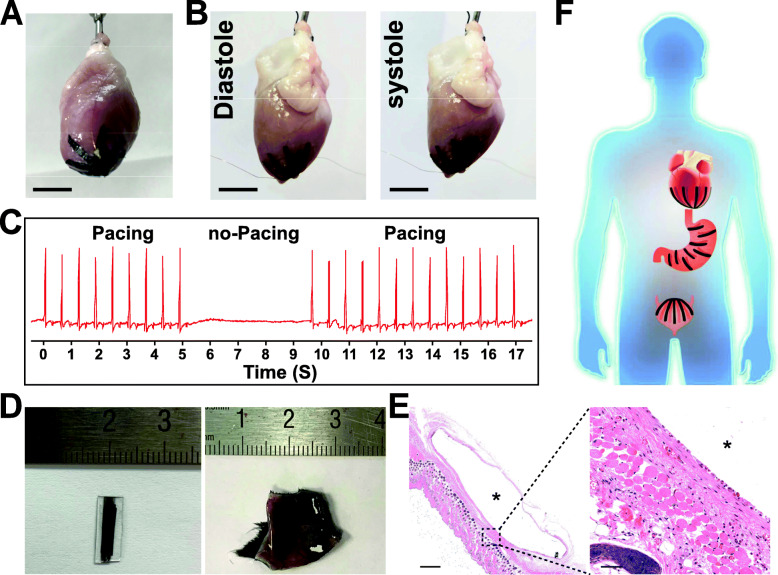
Clinical applications of the MPS. **a** Image showing an MPS device attached on the surface of a Tyrode’s solution perfused rabbit heart, covering the apical portion of the heart. Scale bar, 1 cm. **b** Images indicating that the MPS device dilate (diastole) and contract (systole) along with the beating heart. Scale bars, 1 cm. **c** ECG tracing demonstrating that the MPS device offers a pacing function owe to excellent attachment. **d** MPS appearance shown before implantation (left), and macroscopic view of explanted MPS after 4 weeks of subcutaneous implantation (right). **e** Hematoxylin/eosin staining of tissue section and space (*) left by MPS implant (MPS implant was washed way during staining), after 4 weeks of subcutaneous implantation. Scale bar: 500 μm (left), 50 μm (right). **f** Schematic illustration demonstrating the potential clinical applications of the electrical driven MPS in dynamic organs with failed function, such as heart, stomach and urinary bladder. Scale bars, 1 cm

The clinical applications of the MPS were demonstrated with ex vivo organs. Due to the excellent flexibility and light-weight, the MPS was easily attached directly onto the surface of a Tyrode’s solution perfused rabbit heart, covering the apical portion of the heart where includes both the left and right ventricles (Fig. 5a-b). The electrocardiogram (ECG) of the rabbit heart was monitored in real-time to indicate the beating rhythm of the heart (Fig. 5c and S22, supporting information). When the pacemaker connected MPS device was turned on to output controlled pacing signals, a continuous and regular ECG tracing was observed. This result suggests that the device offers a pacing function owe to excellent attachment, and is capable of contracting and dilating along with the beating heart (Fig. 5b-c). Therefore, the MPS is designed for failed hearts with deteriorative contractility, especially for those hearts accompanied with asynchronous contractions of left and right ventricles.

Finally, this MPS was subcutaneously implanted in mice to assess in vivo biodegradation and local interaction between the implant and the tissue. Implanted MPS was excised for evaluation at 2 weeks and 4 weeks. The shape and size of the implants remain the same after 4 weeks, indicate that the MPS is stable in vivo without structure destruction or biodegradation (Fig. 5d and e, and S23, supporting information). Hematoxylin and eosin staining showed that inflammatory cells increased near the interface of MPS and tissue at 2 weeks, which indicates a postoperative inflammatory response. However, this inflammatory response disappeared at 4 weeks after implantation (Fig. 5e and S23, supporting information). The boundary between the MPS and the tissue is regular and clear, suggesting that the interaction between the MPS and tissue is very mild without noticeable fibrous tissue proliferation or adhesion (Fig. 5e and S23, supporting information). These results indicate that the MPS has excellent biocompatibility and structural stability.

The applications of the electrical driven power assist unit can be further expanded to other dynamic organs with disordered or failed function, such as urinary bladder and stomach (Fig. 5f), to provide rational choice for their future clinical therapeutic scheme. In the future work, optimized actuator devices with stronger contraction strength and bigger contraction amplitude can be further explored. It should be noted, in practical applications, the stronger or bigger contraction does not necessarily lead to better outcomes. The functional characteristics of the device, such as contraction intensity and amplitude, should be carefully customized to match the target organs before clinical transformation.

## Conclusion

In summary, we have demonstrated a CNTs based MPS that is able to provide in situ power assist and synchronous electrical impulse to restore a failed heart. First, the unique interface provided by SA­CNTs was demonstrated to enhance the EMT and direct the migration of pro-regenerative epicardial cells, which are involved in cardiac repair and regeneration. Second, the power assist unit revealed an excellent frequency response to external alternating voltage, as well as natural heart mimicked contractility and amplitude. The flower-shape design provides a strategy to improve the deteriorating cardiac function in a failing heart, as well as to offer a structure support to the thin scar area for a failed heart post-infraction. Moreover, the flexible MPS exhibited an excellent pacing function via direct attachment onto a rabbit heart, which enables the delivery of biventricular pacing signals to correct electrical dyssynchrony. Finally, this device is designed to work at the host heart surface and does not contact blood, thus avoiding the adverse effects of long-term anticoagulant therapy; as well as obviating the destruction of blood cells, serious bleeding and thrombosis complications accompanied with traditional mechanical devices. Therefore, the MPS integrated with cardiac resynchronization pacing, in situ cardiac power assist and cardiac regeneration promotion, reveals a potential in restoring in situ the normal blood-pumping function of a failed heart, especially a failed heart following infarction.

## Supplementary Information

**Additional file 1: Supporting Information. Figure S1.** SEM images of SA-CNTs. A) Low-magnitude and B) high-magnitude SEM images of SA-CNTs. SEM, scanning electron microscope. Scale bar: 100μm (A), Scale bar: 1μm (B). **Figure S2.** A schematic representation of the epicardial outgrowth culture from embryonic mouse hearts. **Figure S3.** Identification of epicardial cells. A, B) Epicardial cells are identified with immunolabeling of A) characteristic mesothelial marker (nuclear Wilms-tumor1, WT1, and B) organized epithelial tight junctions (Zonula occludens-1, ZO1) at day3 of culture. **Figure S4.** Representative images indicate that epicardial outgrow from the embryonic mouse heart to both Coverglass and SA-CNTs can be observed within 24 hours after explant planting, using phase contrast light microscopy. **Figure S5.** A-C) Representative phase contrast light microscopy images indicating that epicardial cells outgrowing from heart explants placed on Coverglass (control) formed a tightly packed sheet of epithelial cells at Day2. B and C): the enlarged image of a selected zone in A. D-F) Epicardial cells from explants placed on SA-CNTs (begin to exhibit a lamellipodia (arrowed) and migratory behavior at Day2. E and F): the enlarged image of a selected zone in D. Scale bars, 500μm. **Figure S6.** Schematic illustration demonstrating the isolation of primary epicardial cells using an outgrowth culture assay and the cells migration manner on different substrates. A, D) E12.5 heart ventricles placed on Epicardial outgrowth on Coverglass (control, A) and SA-CNTs (D). B, E) Epicardial cells outgrowing from heart at day2, epicardial outgrowth from heart explants placed on Coverglass (control) formed a tightly packed sheet of epithelial cells displaying cobblestoned morphology (B), while many cells on SA-CNTs exhibit an a lamellipodia with elongated fibroblast-like shape, particularly those cells at periphery of epithelial sheets (E). C, F) Different cell migration manner on Coverglass and SA-CNTs. At day3, the foremost cells of epicardial outgrowth on Coverglass stuck to the substrate and stopped moving forward, resulting in a formation of “Dam”, until the trapped outgrowing cells accumulate and broke through the “Dam” and poured out (C), in comparison, this phenomenon is rarely observed in the SA-CNTs group, with the peripheral cells of the outgrowth on SA-CNTs migrating far away from the central cells (F). **Figure S7.** Migration patterns of epicardial cells outgrowing from heart explants placed on Coverglass. Typical image after 4 day of culture showing that the cells on Coverglass spread around in a non-directional manner. **Figure S8.** Quantitation of migration distance of epicardial cells grown out from heart explant on control and SA-CNTs after 4 day of culture, showing that epicardial cells on SA-CNTs tend to migrate along the CNT-aligned direction (L_a_), while the cells on Coverglass spread around in a non-directional manner. n = 7 for control sample and n = 13 for SA-CNTs from 5 independent experiments. *p < 0.05. **Figure S9.** Migration patterns of epicardial cells outgrowing from heart explants placed on E-SA-CNTs (SA-CNTs plus external electrical stimulation). Typical image after 4 day of culture showing that the cells on E-SA-CNTs tend to migrate along the CNT-aligned direction (L_a_). **Figure S10.** Biocompatibility evaluation of SA-CNTs on epicardial cells using TUNEL staining. A) Representative confocal images of epicardial cells outgrowing on Coverglass (Control), SA-CNTs and E-SA-CNTs being labelled with TUNEL assay for apoptotic cells (green), and co-stained with DAPI (blue) for cell nuclei. B) Quantitative apoptotic nuclei in total DAPI+ for epicardial cells outgrowing on Coverglass (Control), SA-CNTs and E-SA-CNTs. E-SA-CNTs, SA-CNTs plus external electrical stimulation. n = 6 for each sample. *n.s.*, not significant. **Figure S11.** An image of an MPS in larger size covering the ventricles of a Tyrode's solution perfused rabbit heart. Scale bar: 1cm. **Figure S12.** Cross section SEM images of the electromechanical actuator. A) Low-magnitude and B) high-magnitude of the device without PDMS packing. C) Low-magnitude and D) high-magnitude of the device with PDMS packing. Blue indicates the gel electrolyte area. Scale bar: 25μm. **Figure S13.** SEM images of CNT-film. A) Low-magnitude and B) high-magnitude SEM images of CNT-film. Scale bar: 10μm (A), Scale bar: 1μm (B). **Figure S14.** An actuator petal (0.0036 g) lifting/pushing objects sticked on the top of the petal during deformation. A) Lifting a weight of 0.0170 g. B) Pushing a weight of 0.1271 g. C) Force / Weight Ratio of adult human heart and actuator petal. **Figure S15.** Typical CV curves of one actuator petal at different scan rates (from 1 to 20 V s^-1^) with voltage window of ± 2 V. **Figure S16.** Images of a flower-shaped power assist unit with heart mimicked systolic and diastolic behavior. A and B) At the scan rate of 5 V s^-1^ with the voltage alternating window of ± 2.0 V. C and D) At the scan rate of 5 V s-1 with the voltage alternating window of ± 2.5 V. Contraction ratio (CR) was defined as (end diastolic dimension (EDD) – end systolic dimension (ESD))/ EDD×100 %. **Figure S17.** Images of a flower-shaped power assist unit with heart mimicked systolic and diastolic behavior at different scan rates with voltage window of ± 2.5 V. **Figure S18.** Three-electrode testing system. A) Illustration of the performed method. Working electrode: CNT-film strip. Reference electrode: Ag/AgCl electrode. Counter electrode: Pt wire. Electrolyte: 10% sulfuric acid aqueous solution. B) Typical CV curves of A. **Figure S19.** The computer simulated structure deformation maps of a flower-shaped power assist unit (scan rate: 1V s^-1^, voltage window: ± 2.5 V). The tip area reveals larger position shifting, while the change of position shifting is mainly concentrated in the middle and lower area. Left, top view; Right, bottom view. **Figure S20.** A) Images of the mechanical property test by Instron. B) A typical resulted stress-strain curve. **Figure S21.** The computer simulated stress distribution maps of flower-shaped power assist units with different Yong’s modulus, showing that the bottom of the flower-shaped device has a larger stress with a smaller deformation, while the top part of each petal reveals a small stress with large position shifting. The strength of the petal increases with the augment of its Yong’s modulus, which can be seen from the stress distribution legend of each simulation. For example, when the Yong’s modulus is 267.2 MPa, the green area represents 2.724 e+1 to 5.444 e+1; when the Yong’s modulus is 400.8 MPa, the green area represents 4.086e+1 to 8.165 e+1.**Figure S22.** Schematic illustration demonstrating the setup of ex vivo testing the MPS pacing function. MPS, multifunctional power assist system. **Figure S23.** Hematoxylin/eosin staining of tissue section of Control (A and C, without implantation) and MPS (B and D) group after 2 and 4 weeks of subcutaneous implantation. * indicates the space left by MPS implant (MPS implant was washed way during staining). Arrow indicates the SA-CNTs left after staining. Scale bar: 500 μm (left), 50 μm (right). **Table S1** Table of sequences for upstream and downstream primer for genes analyzed using qRT-PCR

## Data Availability

The datasets used and/or analyzed during the current study are available from the corresponding author on reasonable request.

## Abbreviations

SA-CNTs: Super-aligned carbon nanotubes sheets
EMT: Epithelial-mesenchymal transition
EDPCs: Epicardial-derived progenitor cells
MPS: Multifunctional power assist system
DMEM: Dulbecco’s modified eagle medium
WT1: Nuclear Wilms-tumor1
ZO1: Zonula occludens-1
α-SMA: α-smooth muscle actin
CVD: Chemical vapor deposition
TUNEL: TdT-mediated dUTP nick-end labeling
qRT-PCR: Quantitative real-time polymerase chain reaction
SM-MHC: Smooth muscle myosin heavy chain
TGF-β1: Transforming growth factor β1
CV: Cyclic Voltammetry
CR: Contraction ratio
ECG: Electrocardiogram
