# Supplementary material for: A carbon nanotubes based in situ multifunctional power assist system for restoring failed heart function
Source: BMC Biomed Eng. 2021 Mar 26;3:5. doi: 10.1186/s42490-021-00051-x (PMC7995575; doi:10.1186/s42490-021-00051-x)
Supplement: Supplementary file 2 — Additional file 1: Supporting Information. Figure S1. SEM images of SA-CNTs. A) Low-magnitude and B) high-magnitude SEM images of SA-CNTs. SEM, scanning electron microscope. Scale bar: 100μm (A), Scale bar: 1μm (B). Figure S2. A schematic representation of the epicardial outgrowth culture from embryonic mouse hearts. Figure S3. Identification of epicardial cells. A, B) Epicardial cells are identified with immunolabeling of A) characteristic mesothelial marker (nuclear Wilms-tumor1, WT1, and B) organized epithelial tight junctions (Zonula occludens-1, ZO1) at day3 of culture. Figure S4. Representative images indicate that epicardial outgrow from the embryonic mouse heart to both Coverglass and SA-CNTs can be observed within 24 hours after explant planting, using phase contrast light microscopy. Figure S5. A-C) Representative phase contrast light microscopy images indicating that epicardial cells outgrowing from heart explants placed on Coverglass (control) formed a tightly packed sheet of epithelial cells at Day2. B and C): the enlarged image of a selected zone in A. D-F) Epicardial cells from explants placed on SA-CNTs (begin to exhibit a lamellipodia (arrowed) and migratory behavior at Day2. E and F): the enlarged image of a selected zone in D. Scale bars, 500μm. Figure S6. Schematic illustration demonstrating the isolation of primary epicardial cells using an outgrowth culture assay and the cells migration manner on different substrates. A, D) E12.5 heart ventricles placed on Epicardial outgrowth on Coverglass (control, A) and SA-CNTs (D). B, E) Epicardial cells outgrowing from heart at day2, epicardial outgrowth from heart explants placed on Coverglass (control) formed a tightly packed sheet of epithelial cells displaying cobblestoned morphology (B), while many cells on SA-CNTs exhibit an a lamellipodia with elongated fibroblast-like shape, particularly those cells at periphery of epithelial sheets (E). C, F) Different cell migration manner on Coverglass and SA-C [file 42490_2021_51_MOESM1_ESM.docx]

**Supporting Information**

A carbon nanotubes based in situ multifunctional power assist system for restoring failed heart function

Quanfu Xu^1^, Yuli Yang^1^, Jianwen Hou^1^, Taizhong Chen^1^, Yudong Fei^1^, Qian Wang^1^, Qing Zhou^1^, Wei Li^1^, Jing Ren^2^*, and Yi-Gang Li^1^*

^1^ Department of Cardiology, Xinhua Hospital, School of Medicine, Shanghai Jiao Tong University, Shanghai 200092, China; Email: liyigang@xinhuamed.com.cn

^2^ School of Physical Science and Technology, ShanghaiTech University, 393 Middle Huaxia Road, Shanghai 201210, China; Email: renjing@shanghaitech.edu.cn

***** Correspondence: liyigang@xinhuamed.com.cn (Y.-G.L.); renjing@shanghaitech.edu.cn (J.R.)

**Experimental section**

**Evaluation of epicardial cells behavior**

Embryonic hearts explants and outgrowing epicardial cells on SA-CNTs or Coverglass were evaluated using phase contrast light microscopy connecting to a CCD camera. Images were taken at Days 2, 3, 4, 5, 6, 7 during culturing, and analyzed by ImageJ software. Cell separation was analyzed by measuring the maximal nuclei distance of peripheral cells of epicardial outgrowth explant using immunofluorescence staining (α-SMA) images at Day 6 of culture using the ImageJ software.

**Immunofluorescent staining**

Cells seeded on different substrates were rinsed and fixed in 4% paraformaldehyde (PF) in Dulbecco's phosphate buffered saline (DPBS) for 15 min at room temperature and permeabilized with 0.3% Triton X-100 for 10 min at room temperature. Then, the samples were blocked in 10% goat serum (GS) and were soaked (4 °C, overnight) in primary antibodies, subsequently treated with corresponding secondary antibodies in 10% GS for 60 min at room temperature. The following antibodies were used for immunoﬂuorescence: α-SMA (Mouse, A2547, Invitrogen, Carlsbad, CA, USA)，WT1 (Rabbit, PA5-16879, Invitrogen, Carlsbad, CA, USA), ZO-1 (Rabbit, 40-2200, Invitrogen, Carlsbad, CA, USA)，Cyanine3-conjugated goat anti-mouse antibody (A10521, Invitrogen, Carlsbad, CA, USA) and FITC-conjugated goat anti-rabbit antibody (F-2765, Invitrogen, Carlsbad, CA, USA). Fluorescent images were obtained using a confocal microscopy (Leica TCS SP8, Leica Microsystems, Heidelberg, Germany) and analyzed using ImageJ software.

**Cytotoxicity evaluation**

The cytotoxicity of the different materials was evaluated by terminal deoxynucleotidyl transferase-mediated uridine 5-triphosphatebiotin nick end-labelling (TUNEL) using an *In Situ* Cell Death Detection Kit, Fluorescein (Roche Molecular Biochemicals, Basel, Switzerland) according to manufacturer protocols. Samples were counterstained with 4, 6-diamidino-2-phenyl indole dihydrochloride (DAPI) (blue) for nuclei and mounted in ProLong Gold antifade reagent (Invitrogen, Carlsbad, CA, USA). Apoptotic nuclei were defined as positive when showing a green color under confocal microscopy (Leica TCS SP8, Leica Microsystems, Heidelberg, Germany).

**RNA extraction and real-time qPCR**

Total RNA was isolated from epicardial cells at Day 6 of culture with RNeasy® Mini kits from Qiagen (Hilden, Germany) according to the manufacturer's recommendations. 1000 ng total RNA was reversely transcribed into cDNA using the Prime-Script TM RT reagent kit (Takara, Japan). Quantitative real-time PCR (qRT-PCR) was performed using SYBR green (Takara, Japan) and normalized to GAPDH expression. EMT markers α-SMA, Vimentin and smooth muscle myosin heavy chain (SM-MHC) and EMT inducer TGF-β1 were detected using primer pairs shown in Table S1. The relative expression is calculated using the formula 2^–ΔCt^.


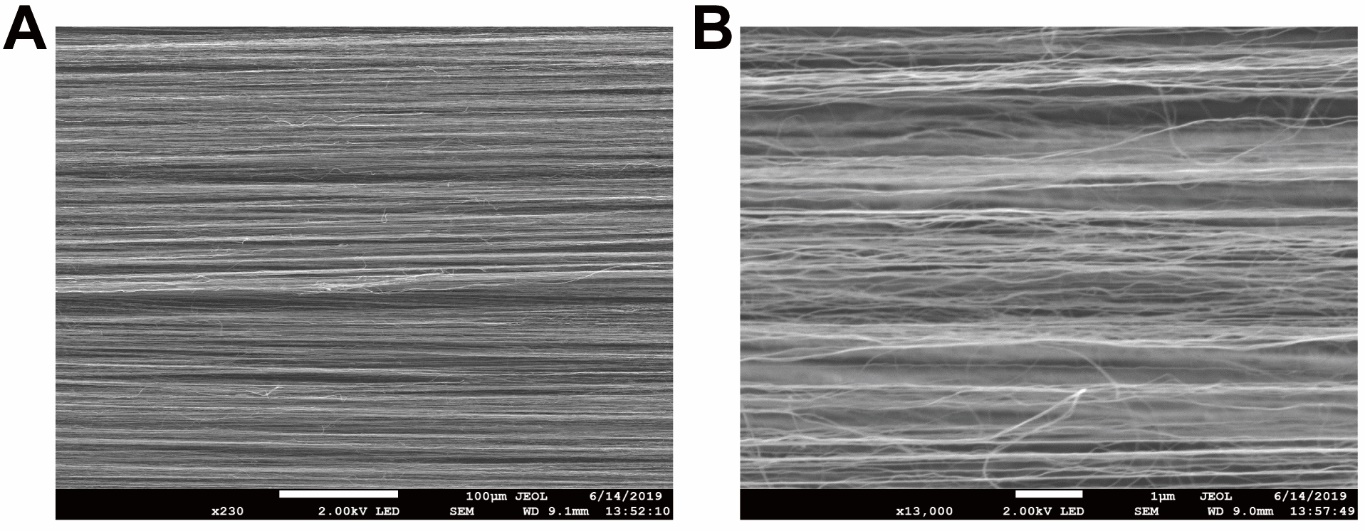


**Figure S1.** SEM images of SA-CNTs. A) Low-magnitude and B) high-magnitude SEM images of SA-CNTs. SEM, scanning electron microscope. Scale bar: 100μm (A), Scale bar: 1μm (B).


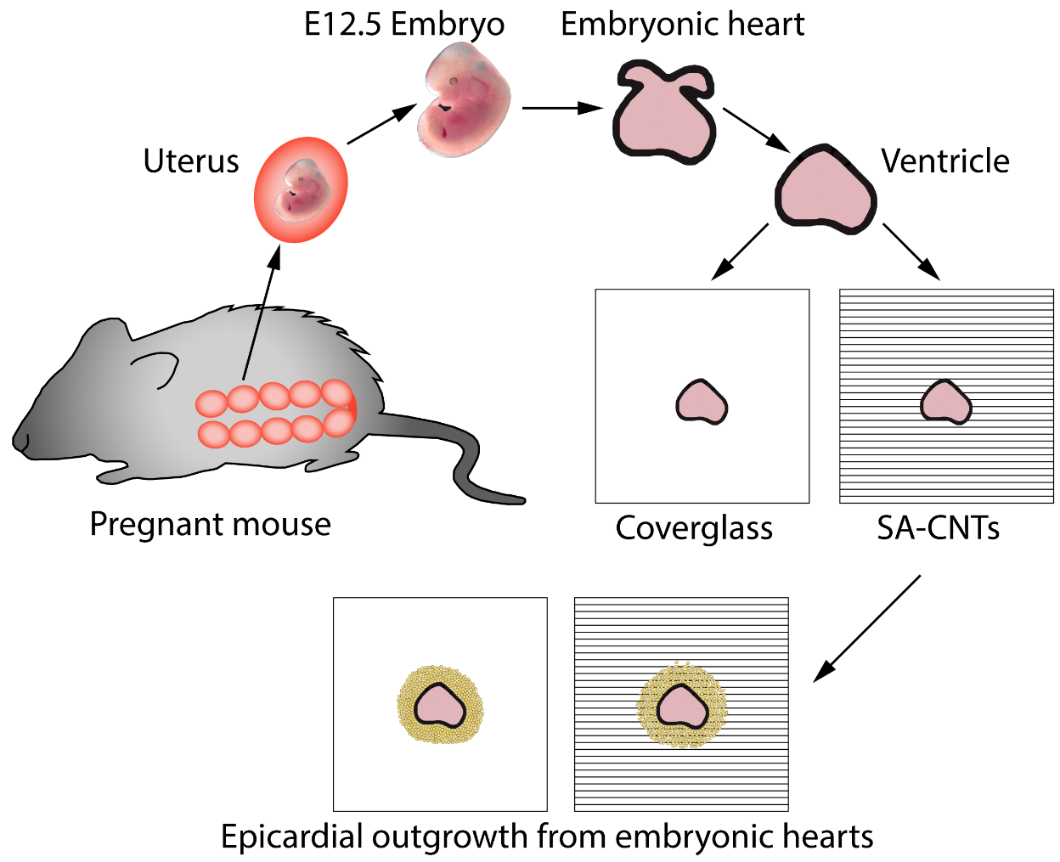


**Figure S2.** A schematic representation of the epicardial outgrowth culture from embryonic mouse hearts.


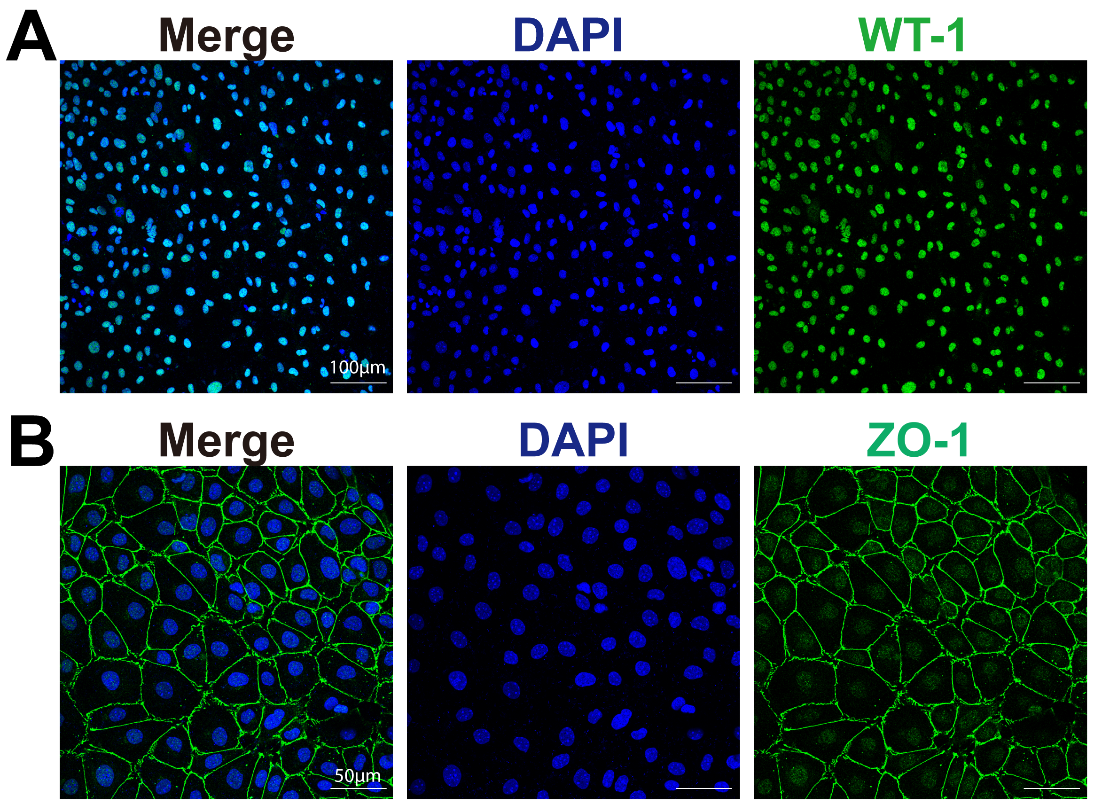


**Figure S3.** Identification of epicardial cells. A, B) Epicardial cells are identified with immunolabeling of A) characteristic mesothelial marker (nuclear Wilms-tumor1, WT1, and B) organized epithelial tight junctions (Zonula occludens-1, ZO1) at day3 of culture.


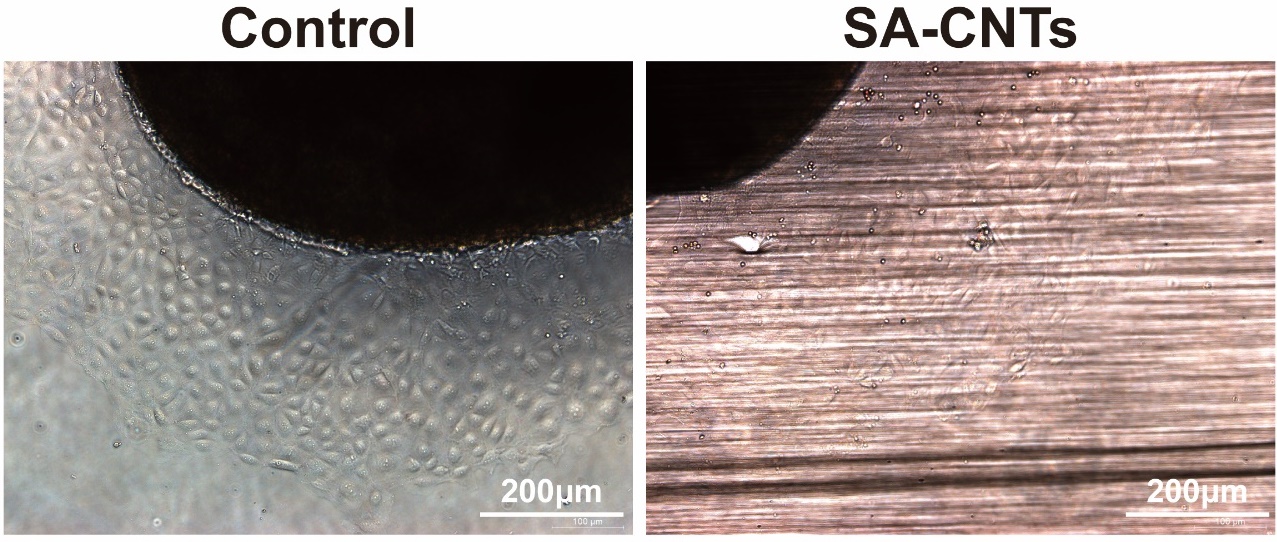


**Figure S4.** Representative images indicate that epicardial outgrow from the embryonic mouse heart to both Coverglass and SA-CNTs can be observed within 24 hours after explant planting, using phase contrast light microscopy.


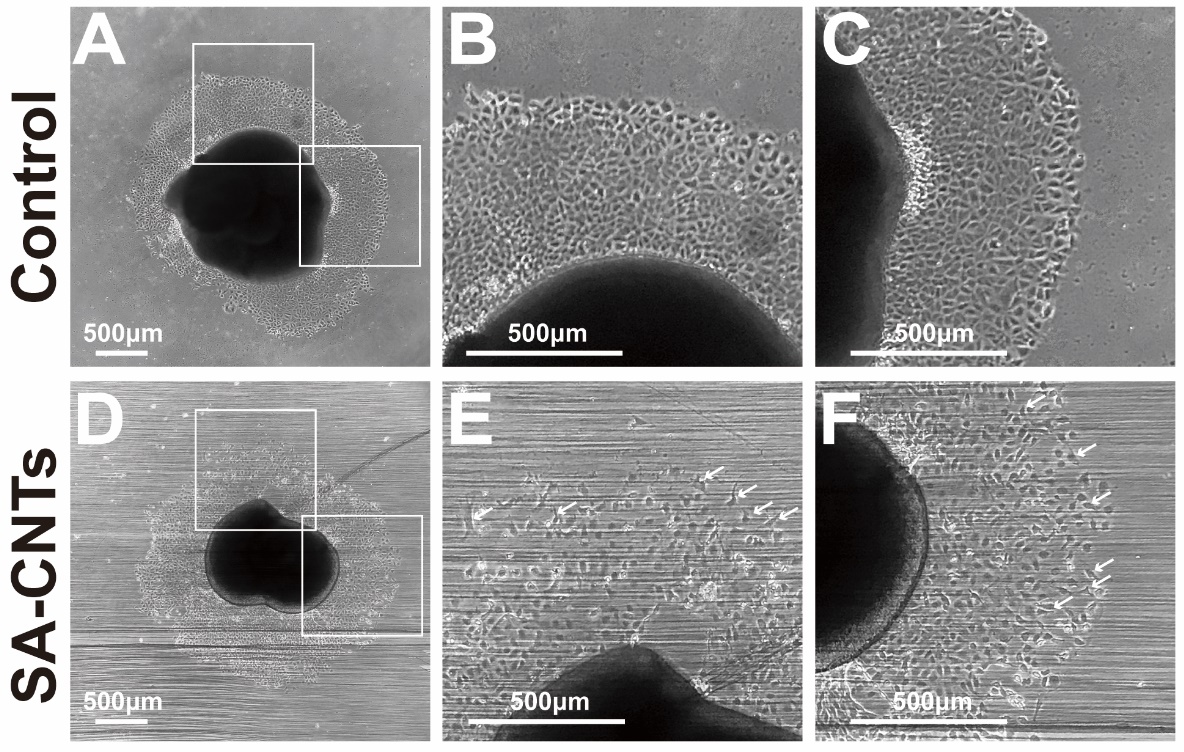


**Figure S5.** A-C) Representative phase contrast light microscopy images indicating that epicardial cells outgrowing from heart explants placed on Coverglass (control) formed a tightly packed sheet of epithelial cells at Day2. B and C): the enlarged image of a selected zone in A. D-F) Epicardial cells from explants placed on SA-CNTs (begin to exhibit a lamellipodia (arrowed) and migratory behavior at Day2. E and F): the enlarged image of a selected zone in D. Scale bars, 500μm.


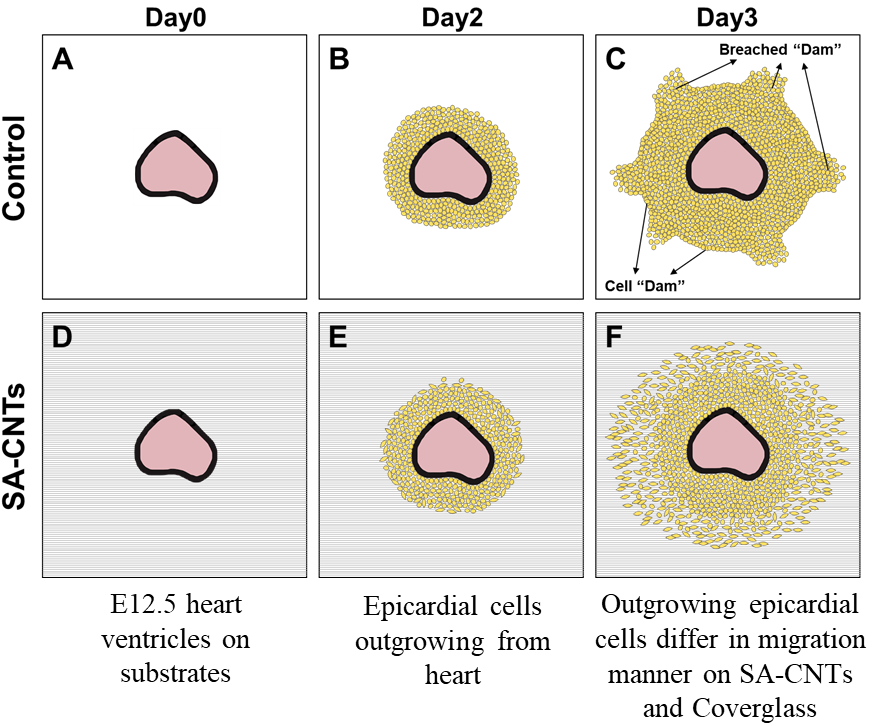


**Figure S6.** Schematic illustration demonstrating the isolation of primary epicardial cells using an outgrowth culture assay and the cells migration manner on different substrates. A, D) E12.5 heart ventricles placed on Epicardial outgrowth on Coverglass (control, A) and SA-CNTs (D). B, E) Epicardial cells outgrowing from heart at day2, epicardial outgrowth from heart explants placed on Coverglass (control) formed a tightly packed sheet of epithelial cells displaying cobblestoned morphology (B), while many cells on SA-CNTs exhibit an a lamellipodia with elongated ﬁbroblast-like shape, particularly those cells at periphery of epithelial sheets (E). C, F) Different cell migration manner on Coverglass and SA-CNTs. At day3, the foremost cells of epicardial outgrowth on Coverglass stuck to the substrate and stopped moving forward, resulting in a formation of “Dam”, until the trapped outgrowing cells accumulate and broke through the “Dam” and poured out (C), in comparison, this phenomenon is rarely observed in the SA-CNTs group, with the peripheral cells of the outgrowth on SA-CNTs migrating far away from the central cells (F).


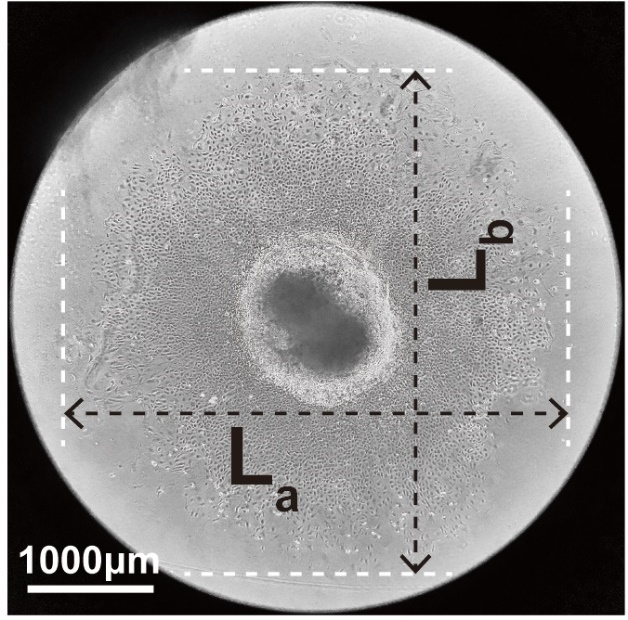


**Figure S7.** Migration patterns of epicardial cells outgrowing from heart explants placed on Coverglass. Typical image after 4 day of culture showing that the cells on Coverglass spread around in a non-directional manner.


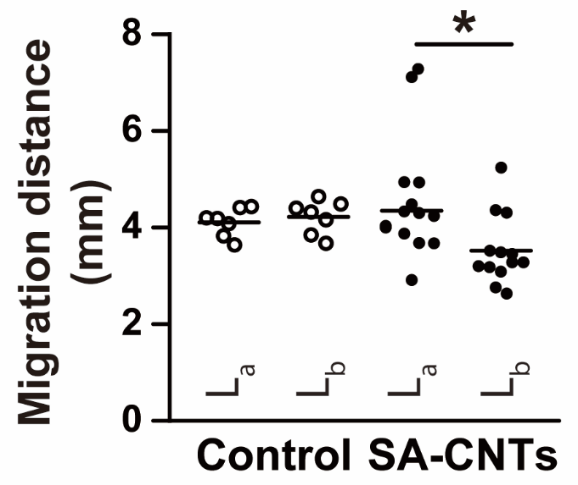


**Figure S8.** Quantitation of migration distance of epicardial cells grown out from heart explant on control and SA-CNTs after 4 day of culture, showing that epicardial cells on SA-CNTs tend to migrate along the CNT-aligned direction (L_a_), while the cells on Coverglass spread around in a non-directional manner. n = 7 for control sample and n = 13 for SA-CNTs from 5 independent experiments. **p* < 0.05.


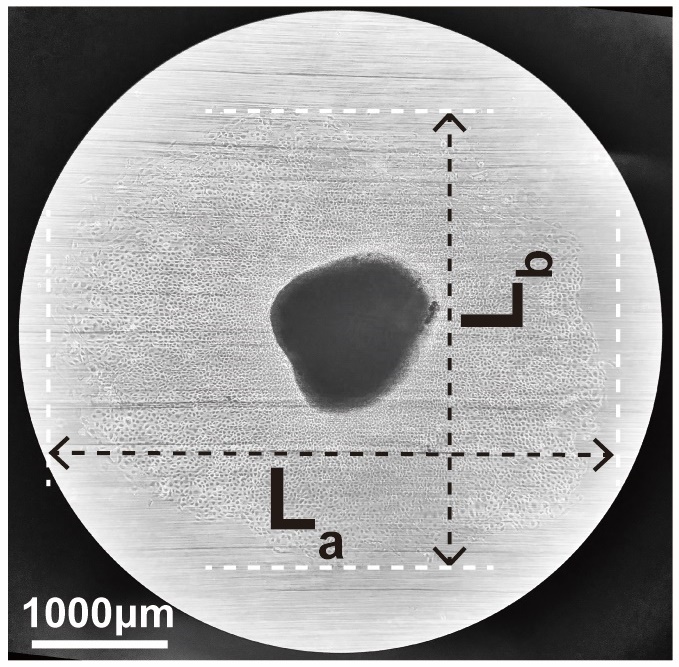


**Figure S9.** Migration patterns of epicardial cells outgrowing from heart explants placed on E-SA-CNTs (SA-CNTs plus external electrical stimulation). Typical image after 4 day of culture showing that the cells on E-SA-CNTs tend to migrate along the CNT-aligned direction (L_a_).


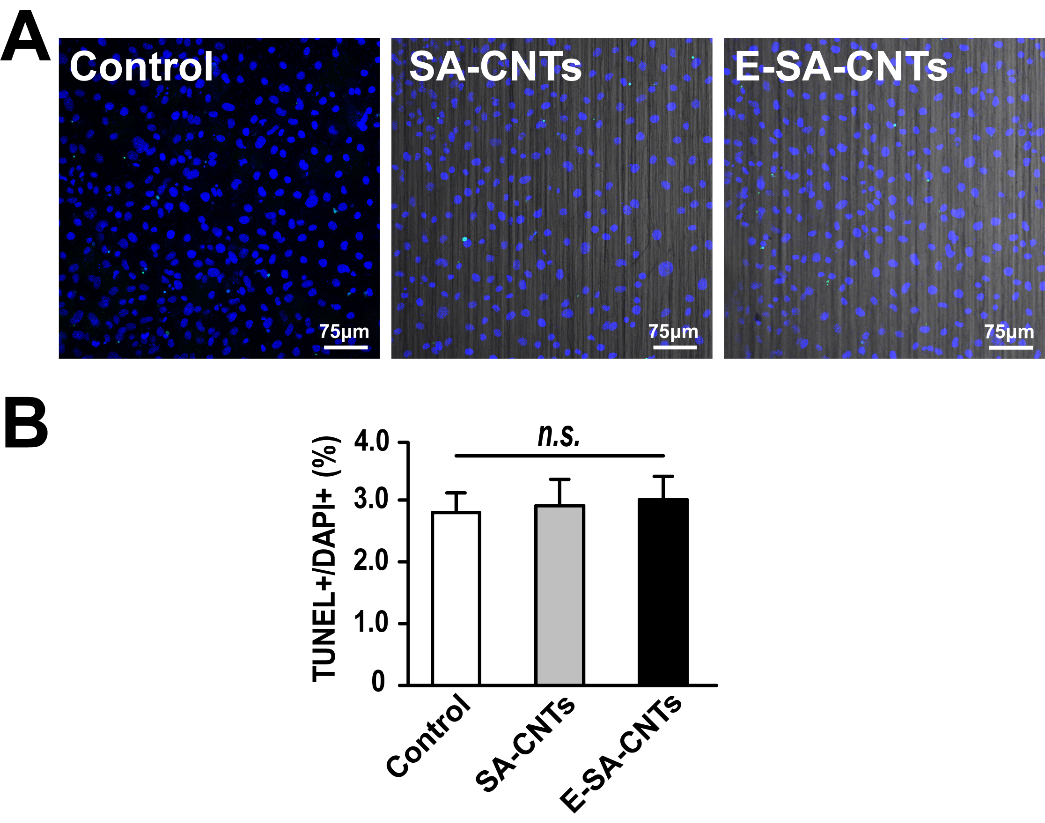


**Figure S10.** Biocompatibility evaluation of SA-CNTs on epicardial cells using TUNEL staining. A) Representative confocal images of epicardial cells outgrowing on Coverglass (Control), SA-CNTs and E-SA-CNTs being labelled with TUNEL assay for apoptotic cells (green), and co-stained with DAPI (blue) for cell nuclei. B) Quantitative apoptotic nuclei in total DAPI+ for epicardial cells outgrowing on Coverglass (Control), SA-CNTs and E-SA-CNTs. E-SA-CNTs, SA-CNTs plus external electrical stimulation. n = 6 for each sample. *n.s.*, not significant.

**Figure S11.** An image of an MPS in larger size covering the ventricles of a Tyrode's solution perfused rabbit heart. Scale bar: 1cm.


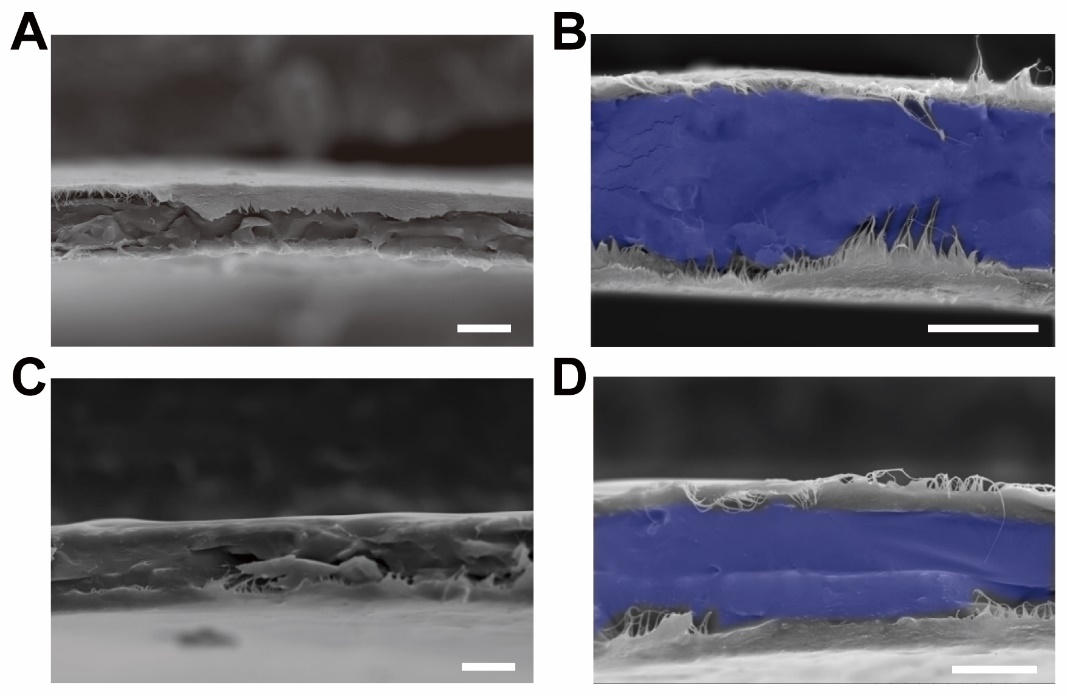


**Figure S12.** Cross section SEM images of the electromechanical actuator. A) Low-magnitude and B) high-magnitude of the device without PDMS packing. C) Low-magnitude and D) high-magnitude of the device with PDMS packing. Blue indicates the gel electrolyte area. Scale bar: 25μm.


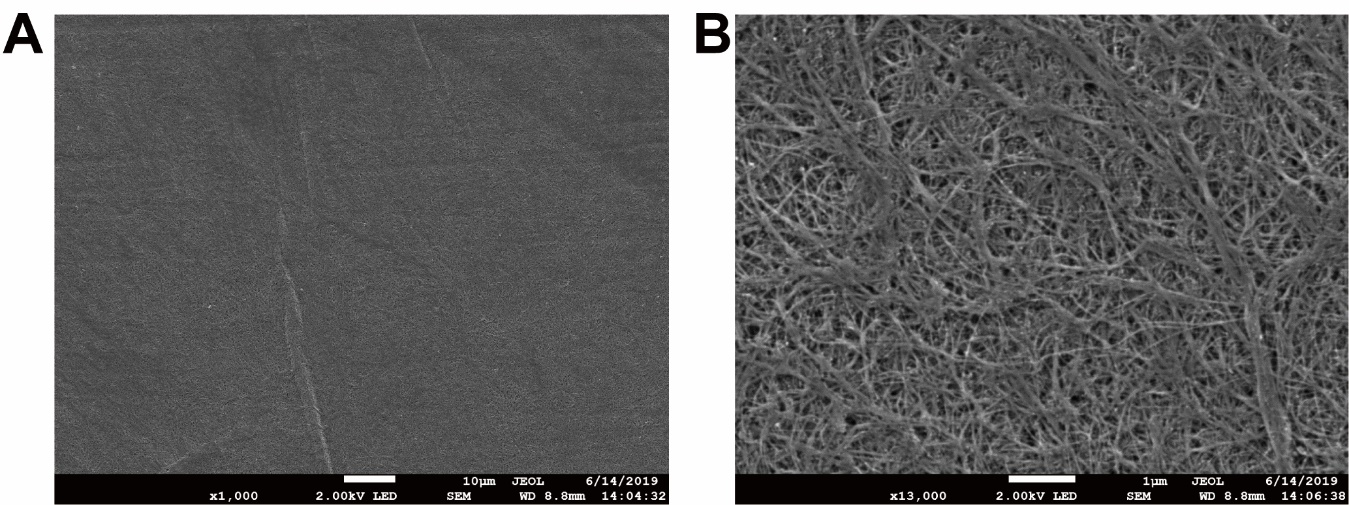


**Figure S13.** SEM images of CNT-film. A) Low-magnitude and B) high-magnitude SEM images of CNT-film. Scale bar: 10μm (A), Scale bar: 1μm (B).

**Figure S14.** An actuator petal (0.0036 g) lifting/pushing objects sticked on the top of the petal during deformation. A) Lifting a weight of 0.0170 g. B) Pushing a weight of 0.1271 g. C) Force / Weight Ratio of adult human heart and actuator petal.

**Figure S15.** Typical CV curves of one actuator petal at different scan rates (from 1 to 20 V s^-1^) with voltage window of ± 2 V.


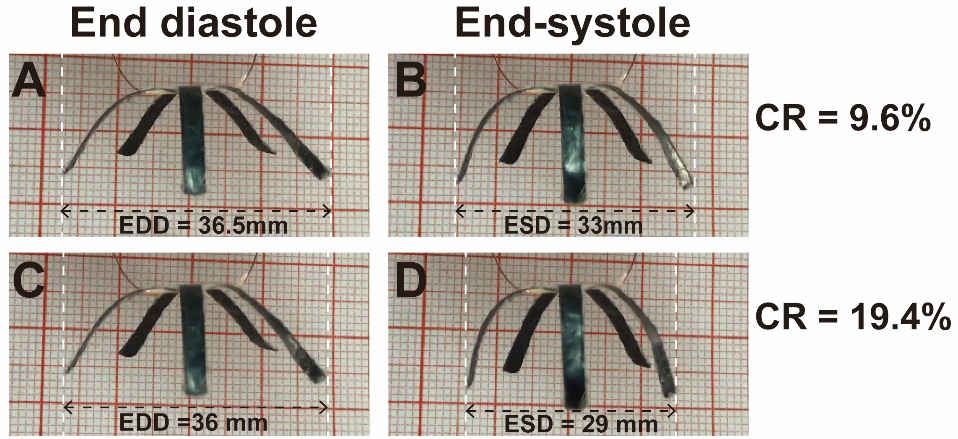


**Figure S16.** Images of a flower-shaped power assist unit with heart mimicked systolic and diastolic behavior. A and B) At the scan rate of 5 V s^-1^ with the voltage alternating window of ± 2.0 V. C and D) At the scan rate of 5 V s-1 with the voltage alternating window of ± 2.5 V. Contraction ratio (CR) was defined as (end diastolic dimension (EDD) – end systolic dimension (ESD))/ EDD×100 %.


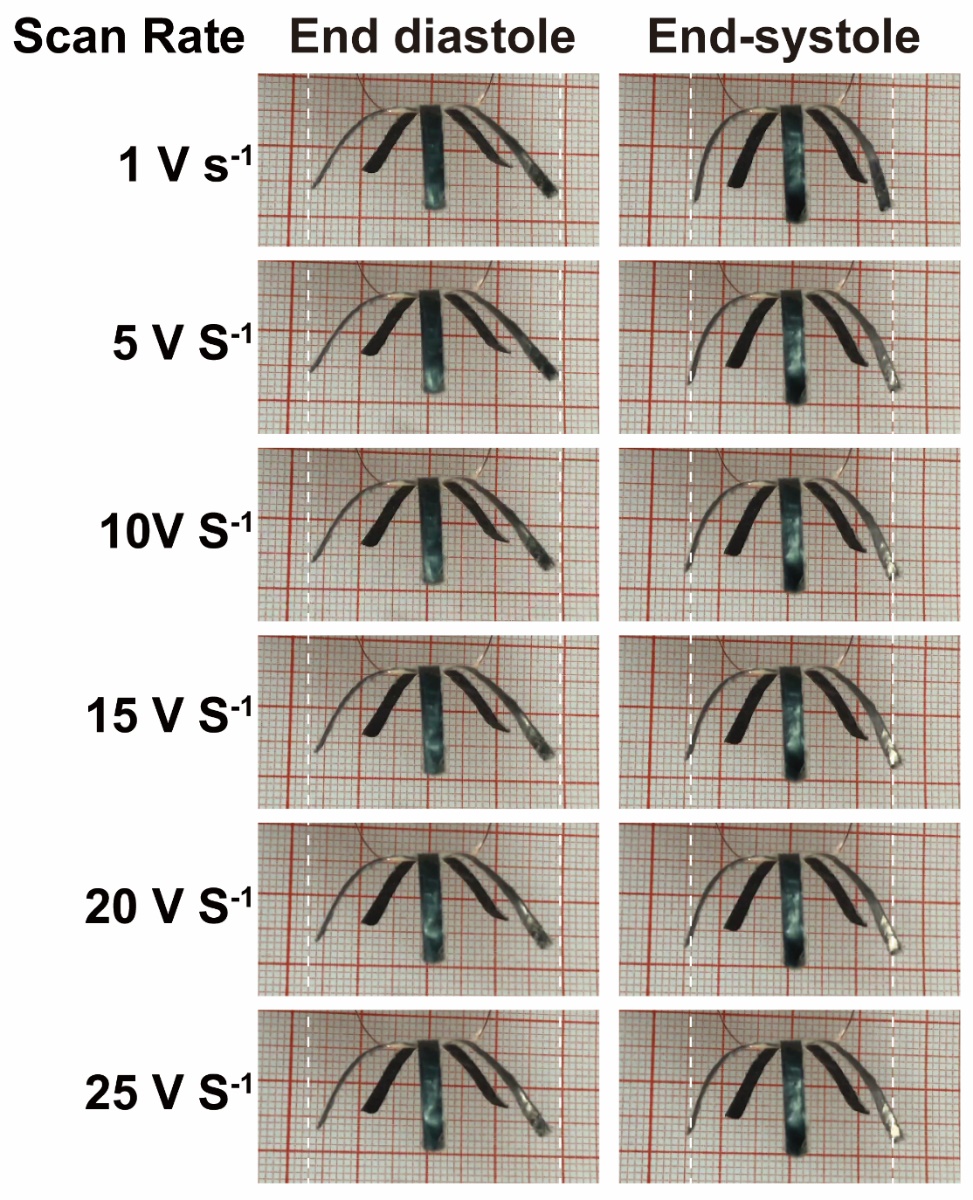


**Figure S17.** Images of a flower-shaped power assist unit with heart mimicked systolic and diastolic behavior at different scan rates with voltage window of ± 2.5 V.

**Figure S18.** Three-electrode testing system. A) Illustration of the performed method. Working electrode: CNT-film strip. Reference electrode: Ag/AgCl electrode. Counter electrode: Pt wire. Electrolyte: 10% sulfuric acid aqueous solution. B) Typical CV curves of A.


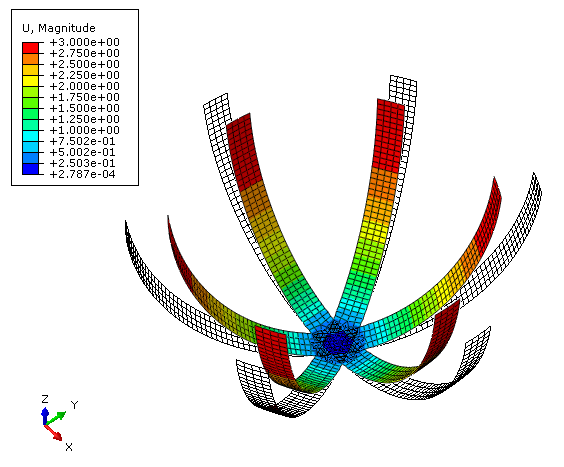

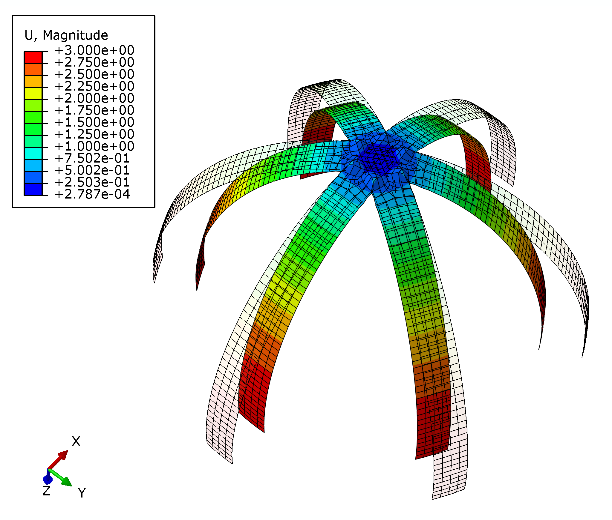


**Figure S19.** The computer simulated structure deformation maps of a flower-shaped power assist unit (scan rate: 1V s^-1^, voltage window: ± 2.5 V). The tip area reveals larger position shifting, while the change of position shifting is mainly concentrated in the middle and lower area. Left, top view; Right, bottom view.


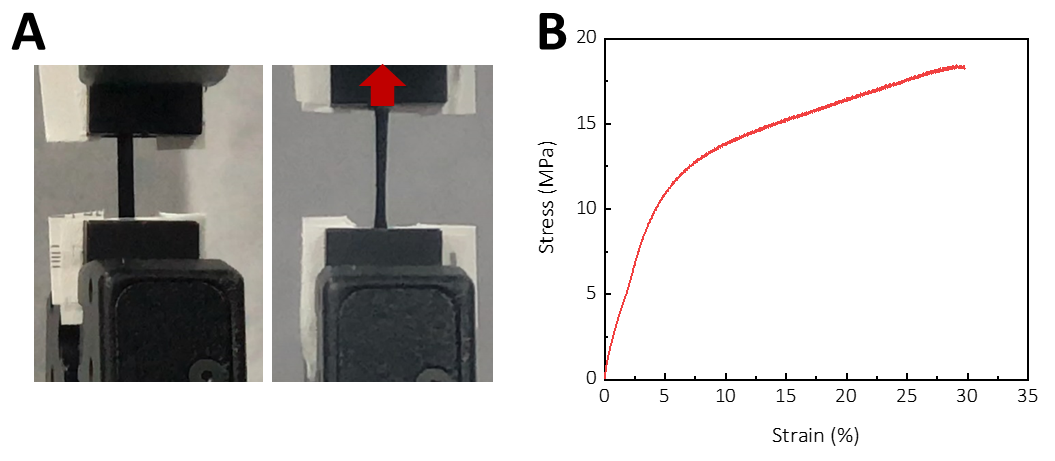


**Figure S20.** A) Images of the mechanical property test by Instron. B) A typical resulted stress-strain curve.

**Figure S21.** The computer simulated stress distribution maps of flower-shaped power assist units with different Yong’s modulus, showing that the bottom of the flower-shaped device has a larger stress with a smaller deformation, while the top part of each petal reveals a small stress with large position shifting. The strength of the petal increases with the augment of its Yong’s modulus, which can be seen from the stress distribution legend of each simulation. For example, when the Yong’s modulus is 267.2 MPa, the green area represents 2.724 e+1 to 5.444 e+1; when the Yong’s modulus is 400.8 MPa, the green area represents 4.086e+1 to 8.165 e+1.

**Figure S22.** Schematic illustration demonstrating the setup of ex vivo testing the MPS pacing function. MPS, multifunctional power assist system.


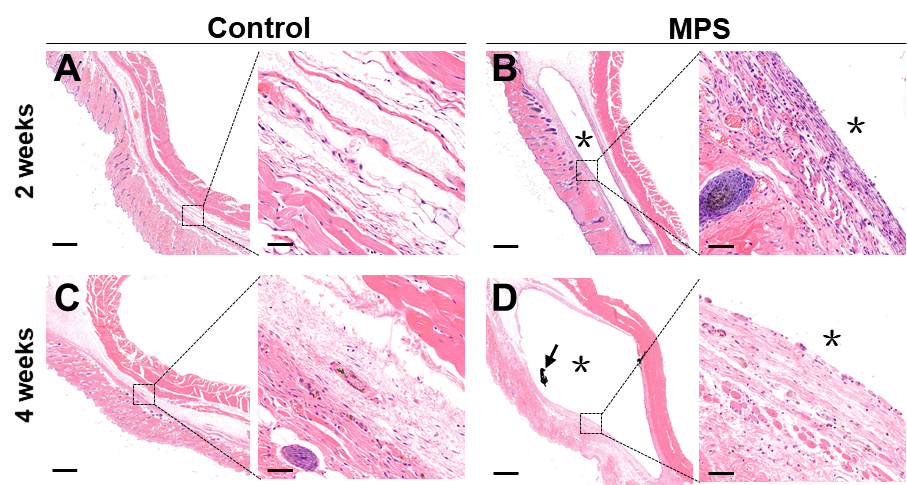


**Figure S23.** Hematoxylin/eosin staining of tissue section of Control (A and C, without implantation) and MPS (B and D) group after 2 and 4 weeks of subcutaneous implantation. **＊**indicates the space left by MPS implant (MPS implant was washed way during staining). Arrow indicates the SA-CNTs left after staining. Scale bar: 500 μm (left), 50 μm (right).

**Table S1** Table of sequences for upstream and downstream primer for genes analyzed using qRT-PCR

| Gene (mouse) | Forward Primer | Reverse Primer |
| --- | --- | --- |
| α-SMA | GTCCCAGACATCAGGGAGTAA | TCGGATACTTCAGCGTCAGGA |
| smMHC | GCCGGAAAGACAGAGAACAC | GGATTGGGTTTGCCTGTAGA |
| Vimentin | CGTCCACACGCACCTACAG | GGGGGATGAGGAATAGAGGCT |
| GAPDH | ACCACAGTCCATGCCATCAC | CACCACCCTGTTGCTGTAGCC |
| TGF-β1 | CTCCCGTGGCTTCTAGTGC | GCCTTAGTTTGGACAGGATCTG |

α-SMA, α smooth muscle actin; smMHC, smooth muscle myosin heavy chain; GAPDH, glyceraldehyde-3-phosphate dehydrogenase and TGF-β1, transforming growth factor beta 1.

**Supporting Video**

**Video S1.** Video of a flower-shaped power assist unit performing heart mimicked systolic-diastolic behavior with increased scan rates at voltage window of ± 2.5 V.
